# Wide QRS tachycardia after recent pulmonary vein isolation

**DOI:** 10.1007/s12471-021-01547-w

**Published:** 2021-02-15

**Authors:** R. S. Prisecaru, V. Costache

**Affiliations:** 1 European Hospital Polisano, Medlife Sibiu, Sibiu, Romania; 2 Lucian Blaga University, Sibiu, Romania

**Fig. 1:**
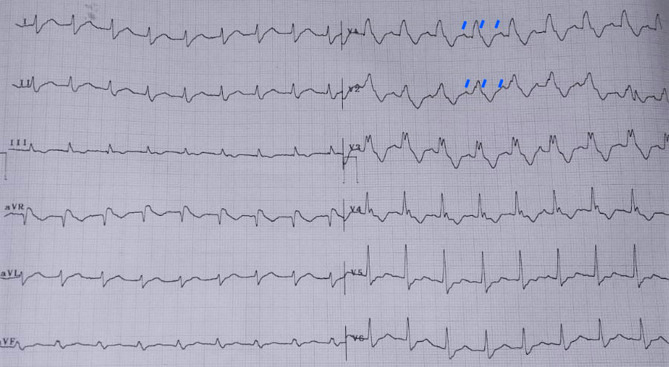
Electrocardiogram shows a wide QRS tachycardia with right bundle branch block morphology. P waves are visible in precordial leads with 2:1 conduction, compatible with atrial tachycardia and aberrant conduction

A 48-year-old male was admitted to our hospital for palpitations. The electrocardiogram obtained on admission is shown in Fig. 1. Echocardiography and other testing revealed no abnormalities. Eight weeks earlier, he had undergone pulmonary vein isolation for paroxysmal atrial fibrillation. At discharge, he was started on treatment with anticoagulant, beta blocker and a Class 1C antiarrhythmic drug, flecainide.

Class 1C antiarrhythmic drugs, such as flecainide and propafenone, depress cardiac excitability and decrease conduction throughout the cardiac tissue due to use-dependent sodium channel blockade [1]. Because of slowed conduction through the His-Purkinje system [2], which is more pronounced at faster heart rates, class 1C antiarrhythmic agents can cause a rate-related aberrant conduction of supraventricular tachycardia [3], including atrial flutter with 2:1 conduction, that can mimic ventricular tachycardia.

Distinction is paramount to treatment and prognosis. The patient underwent a second procedure with mitral isthmus radiofrequency ablation and has had no palpitations recurrence since.
